# Surface Modified Nanocellulose and Its Reinforcement in Natural Rubber Matrix Nanocomposites: A Review

**DOI:** 10.3390/polym13193241

**Published:** 2021-09-24

**Authors:** Nik Muhammad Faris Hakimi, Seng Hua Lee, Wei Chen Lum, Siti Fatahiyah Mohamad, Syeed SaifulAzry Osman Al Edrus, Byung-Dae Park, Anis Azmi

**Affiliations:** 1 Institute of Tropical Forestry and Forest Product, Universiti Putra Malaysia, Serdang 43400, Malaysia; nikfaris16.95@gmail.com; 2 Institute for Infrastructure Engineering and Sustainable Management (IIESM), Universiti Teknologi MARA, Shah Alam 40450, Malaysia; anisazmi8855@gmail.com; 3 Radiation Processing and Technology Division, Malaysia Nuclear Agency, Kajang 43000, Malaysia; fatahiyah@nuclearmalaysia.gov.my; 4 Department of Wood and Paper Science, Kyungpook National University, Daegu 41566, Korea; byungdae@knu.ac.kr

**Keywords:** nanomaterials, surface modification, latex, lignocellulosic fibers, conventional fillers

## Abstract

Natural rubber is of significant economic importance owing to its excellent resilience, elasticity, abrasion and impact resistance. Despite that, natural rubber has been identified with some drawbacks such as low modulus and strength and therefore opens up the opportunity for adding a reinforcing agent. Apart from the conventional fillers such as silica, carbon black and lignocellulosic fibers, nanocellulose is also one of the ideal candidates. Nanocellulose is a promising filler with many excellent properties such as renewability, biocompatibility, non-toxicity, reactive surface, low density, high specific surface area, high tensile and elastic modulus. However, it has some limitations in hydrophobicity, solubility and compatibility and therefore it is very difficult to achieve good dispersion and interfacial properties with the natural rubber matrix. Surface modification is often carried out to enhance the interfacial compatibilities between nanocellulose and natural rubber and to alleviate difficulties in dispersing them in polar solvents or polymers. This paper aims to highlight the different surface modification methods employed by several researchers in modifying nanocellulose and its reinforcement effects in the natural rubber matrix. The mechanism of the different surface medication methods has been discussed. The review also lists out the conventional filler that had been used as reinforcing agent for natural rubber. The challenges and future prospective has also been concluded in the last part of this review.

## 1. Introduction

**Figure 1:**
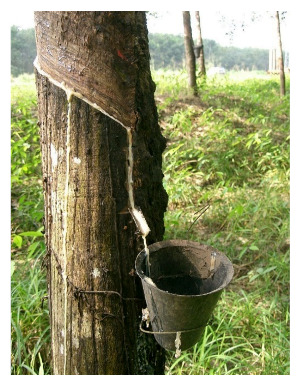
Latex being collected from a tapped rubber tree (PRA (2007) Récolte du latex sur un hévéa au Cameroun. Freely redistribute under Creative Commons Attribution-Sharealike 3.0 Unported License. Figure available from https://commons.wikimedia.org/wiki/File:Latex_-_Hevea_-_Cameroun.JPG [4]).

Natural rubber is a distinctive biopolymer of significant economic importance thanks to its high molecular weight and many others minor components that are existing in the latex. Figure 1 displays the latex (white colored colloidal suspension of rubber particles) being collected from a Para rubber tree (*Hevea brasiliensis*). The rubber tree is still the main source of natural latex as it produces a large amount of high molecular weight latex. Natural rubber could be produced from the latex found in more than 2500 plant species, guayule (*Parthenium argentatum* Gray) and Russian dandelion (*Taraxacum koksaghyz*) being the most promising alternatives to rubber trees [1]. Up until now, natural rubber is still irreplaceable by any other synthetic materials thanks to its resilience, elasticity, abrasion and impact resistance, efficient heat dispersion and malleability at cold temperature [2,3]. 

**Figure 2:**
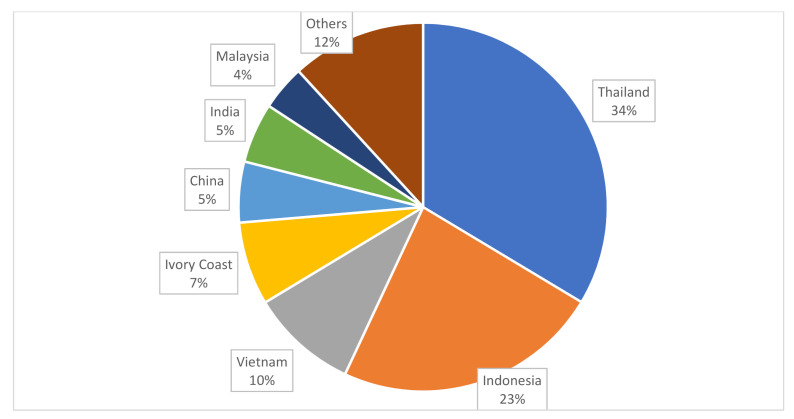
Natural rubber producing countries worldwide in 2020 (Statista 2021).

Natural rubber is an important commodity, particularly to those of the people in the Southeast Asian region. In 2020, the total natural rubber production amounted to 13.008 million metric tons. Asia is the leading natural rubber producing continent worldwide where Thailand, Indonesia, Vietnam, China, India and Malaysia are among the main producers of natural rubber (Figure 2). Around 81% of natural rubber is produced by these six countries with Thailand being the leading producer followed by Indonesia [5]. With very high cost performance, natural rubber becomes a very versatile material that is being used in the production of more than 40,000 products. The most common products include surgical gloves and tires. Automobiles is the leading industry that drives the global market of natural rubber. According to the report by Mordor Intelligence [6], in the year of 2019, automobile tires made up to 46% of the total industrial applications of natural rubber worldwide, followed by footwear (17%), tubes (15%), latex (10%) and others (12%). The demand of natural rubber keeps increasing over years, corresponding to the increased applications of natural rubber. Expert Market Research [7] forecasts the demand for natural rubber is expected to grow at a compound annual growth rate (CAGR) rate of 4.8% in the period of 2021–2026. By 2026, a whopping volume of 20.1 million metric tons is anticipated in order to cater to the demand worldwide. 

Natural rubber is one of the most significant elastomers due to its versatility and application volume. Unfortunately, natural rubber has been identified with some drawbacks such as low modulus and strength. To account for that, crosslinking and adding reinforcing fillers of various sources and aggregate size/aspect ratio, such as cellulose nanocrystals, can improve and modify the mechanical characteristics of natural rubber. Carbon nanotubes, ceramics, and natural fibers are some examples of nanoparticles that can provide polymeric matrices with particular characteristics. When nanofiller is added to a matrix, it can change its mechanical characteristics and its crystallinity and permeability [8]. In the past few decades, natural rubber composites reinforced with various fillers such as lignocellulosic fibers and nanocellulose in replacing non-renewable carbon black has been the main focus of researchers worldwide [9]. In recent years, the usage of nano sized cellulose in reinforcing natural rubber has gained the most attention of researchers. However, the surface characteristic of the nanocellulose is one of the major challenges as it greatly affects the dispersion and interfacial properties of the resultant natural rubber nanocomposite. On that account, surface modification is often employed to modify the nanocellulose in order to achieve better dispersion and interfacial properties with the natural rubber matrix. 

Some reviews have been conducted by several researchers on the topic. The most recent review by Low et al. [10] discussed the recent developments in nanocellulose reinforced rubber matrix composites. Property enhancement of rubber composites (natural rubber and synthetic rubber) as a result of nanocellulose reinforcement has been highlighted. Kargarzadeh et al. [11] reviewed the recent developments in nanocellulose reinforced polymer nanocomposites where the topic was not confined only to nanocellulose reinforced rubber. Nanocellulose reinforced thermoset composites such as epoxy, unsaturated polyester, polyethylene terephthalate (PET), phenol-, melamine-, and urea-formaldehyde resins have also been highlighted in the review. On the other hand, Zhou et al. [9] compiled a review on the rubber composites reinforced with various wood flour and lignocellulosic fibers including oil palm, hemp, husk, bamboo, bagasse, jute etc. However, a limited number of studies on nanocellulose reinforced rubber nanocomposites were reported at the time of writing. A book chapter by Nunes [12] also focused on the rubber nanocomposites with nanocellulose where the structure and properties of different rubber nanocomposites such as natural rubber, epoxidized natural rubber, polybutadiene rubber, ethylene propylene diene methylene rubber and so on were discussed. However, there is still a need for a compilation emphasizing on the surface modification of the nanocellulose and its resultant natural rubber composites. Therefore, this review focuses on summarizing the recent surface modification methods employed on the nanocellulose and its applications in the production of natural rubber nanocomposites. The conventional fillers used in the natural rubber based nanocomposite are also discussed. Some challenges and future perspectives are also highlighted at the last part of this review. 

## 2. Conventional Fillers for Natural Rubber Nanocomposite

Natural rubber is traditionally incorporated with carbon black and silica during the vulcanization process to form rubber composite with enhanced properties. Despite their capacities as rubber composite reinforcements, carbon black and silica are not bio-degradable and require significant energy to manufacture. Carbon black being a petrochemical based product is also non-renewable. Therefore, in the route to search for a renewable and environmentally friendly filler, lignocellulosic fiber has gained attention and traction in the rubber composite industry due to its natural properties. However, the fundamental problem of the lignocellulosic fiber reinforced rubber composite is the poor compatibility of the hydrophilic wood flour or other natural fibers with the hydrophobic rubber matrix, which results in the composite’s mediocre mechanical qualities. In order to improve the compatibility between natural rubber matrix and the filler used, various modification approaches of the fillers used are often conducted. In recent years, the advancement of nanotechnology has propelled surface modified nanocellulose as a viable filler for rubber composite. The role of nanocellulose as a viable filler for rubber composite will be discussed in detailed in the next section. In this section, the main findings of natural rubber composite reinforced with conventional filler (i.e., carbon black, silica and lignocellulosic fibers) from available literatures are discussed and summarized in Table 1, Table 2 and Table 3.

### 2.1. Carbon Black

Table 1 summarizes the main findings of natural rubber composite reinforced with conventional carbon black filler from available literatures. According to Table 1, most researchers studied the effects of types of carbon black, the concentration of carbon black and also the treatment employed to the carbon black used to enhance the curing characteristic and mechanical properties of rubber hybrid composites.

Generally, the application of carbon black filler increases the curing time of the natural rubber composite as shown by the lower cure rate index (CRI). It was also found that the tensile strength of natural rubber composite peaked at 40 phr carbon black content and then decreased with higher carbon black content. However, the modulus increased proportionally with the filler concentration [14,15]. 

Sivaselvi et al. [13] studied the effect of carbon black ratio in hybrid filler on the mechanical properties of rubber composite. The authors concluded that the rubber composite reinforced with the highest carbon black content exhibited the best abrasion resistance index (67%) and tensile and tear strength. This can be explained by the good bonding of the rubber matrix provided by the carbon black particles. The small particle size of carbon black blended well with natural rubber and did not hinder the vulcanization process therefore impart good mechanical properties. Thus, the high carbon black incorporated samples had improved mechanical properties. However, the authors noted that to achieve the optimum mechanical properties in every aspect, a perfect ratio of concentration of fillers is important. Salaeh and Nakason [14] conducted an experiment to study the effects of the types of carbon black filler, high-abrasion furnace (HAF) and extra conductive furnace (ECF) on the curing characteristic and tensile strength of natural rubber composite. They discovered that because ECF has a finer structure than HAF, it absorbs more curing agent and accelerator molecules during vulcanization, delaying the crosslinking reaction. They also concluded that a high concentration level of carbon black content of 50 phr reduced the tensile strength of rubber composite. This could be because a high carbon black content reduces the volume fraction of the rubber component that forms the composite’s continuous matrix [16].

### 2.2. Silica

Table 2 summarizes the main findings of natural rubber composite reinforced with conventional silica-based filler from available literatures. According to Table 2, most researchers studied the effects of type of silica, loadings of silica and also treatment of the silica used on the curing characteristic and mechanical properties of rubber hybrid composites.

**Table 2:** The summary of literatures using silica as main fillers for rubber composite.

Filler/Source	Treatments	Variables	Properties Tested and Findings	References
Silica fume	-	Silica loading (10, 20, 30, 40, 50 phr)	**Curing Characteristic** The cure time of all the reinforced composites increased with the increase in silica fume loading **Tensile Strength and Modulus** Composite added with 20 phr silica showed improved tensile strength. The tensile strength dropped slightly with further increase of silica fume loading. However, the composite’s modulus increased proportionally with silica fume loading. **Tear Strength** Tear strength increased up to 20 phr of silica fume loading and then decreased with further increases in loading.	[17]
Silica, Silica-graphene oxide (SiO_2_@GO)	-	Silica loading of 3 phr	**Tensile strength, Modulus and Elongation at Break** Composite added with silica exhibited insignificant change in tensile strength due to the low content of silica used. However, composite consisted of hybrid silica-graphene oxide filler, showed an increase in tensile strength, elongation at break, and modulus.	[18]
Silica from sugarcane bagasse ash	Drying technique	Freeze drying (FD) and heat drying (HD)	**Curing Characteristic** Scorch time of the rubber composites increased with an increase in silica content. **Tensile Strength** Tensile strength of the tested composites increased with increase in silica content but tended to reduce slightly at higher silica content. **Modulus, Hardness and Elongation at Break** The modulus and hardness of rubber composite increased while elongation at break decreased with an increase in silica content	[19]
Silica, styrene	-	Particle size	**Tensile Strength, Modulus and Hardness** All the mechanical properties of reinforced natural rubber increased compared to rubber compound without reinforcement.	[20]
Silica -precipitated silica (PS), autonomous monodisperse silica (AS)	Silane treatment	Silica dimension and polydispersity	**Tensile Strength and Abrasive Resistance** PS reinforced rubber showed better tensile strength and abrasive resistance than AS reinforced rubber.	[21]
Silica from rice husk	Alkali treatment	Silica loading of 60 phr	**Curing Characteristic** The result shows that the curing time of the rubber composites decrease with increasing silica loading. **Tensile Strength** The tensile strength was determined at the break point of the specimen. Results show the addition of silica in natural rubber matrix resulted in the improvement in the tensile properties **Tear Strength and Hardness** The addition of silica as filler increases the tear strength as well as the hardness of the rubber composite as the silica loading increases.	[22]
Precipitated silica (PSi) and fly ash silica (FASi)	-	Silica loading of 0–75 phr	**Curing Characteristic** The cure time and minimum and maximum torques of the rubber compounds were unaffected by silica loadings from 0 to 30 phr, and above these concentrations, the values progressively increased with increasing PSi loading but the FASi remained the same. 0 to 30 phr FASi could be recommended to natural rubber but not styrene–butadiene rubber **Tensile Strength and Elongation at Break** The tensile strength and elongation at break of rubber composite filled with untreated silica decreased with increasing silica content.	[23]

Table 2 shows that the curing time of rubber composites is affected by the type of silica filler and the amount of silica applied. According to certain researches, the higher the silica level, the longer the curing period. Other researches, on the other hand, found the opposite. The mechanical properties of silica reinforced rubber composite are more uniform among studies and show a similar trend to carbon black reinforced rubber composite. The addition of silica increases the mechanical properties of rubber composite compared to the rubber composite without reinforcement. However, the strength of the material is not positively proportionate to the silica content added. The strength peaks at certain silica content before it decreases as more silica is added.

The curing characteristic of rubber composite is influenced by the filler content. Boonmee and Jarukumjor [19] noted that the scorch time of natural composites was increased with an increase in silica nanoparticle content. Dileep et al. [17] also reported similar findings, which stated that at higher loading of silica fume the curing time increased. This might be cause by the disturbance of vulcanization process by silica particles surface. Sombatsompop et al. [23] found out that the cure time of rubber composite was not affected by the silica concentration below 15 phr but decreased abruptly at further concentration. At lower silica concentration, the silica particles were separated from one another, forming a dispersed gel through the rubber matrix without hindering the polymerization process. This in agreement with results obtain by Ahmed et al. [22] which observed that curing time decreased with an increase in silica loading.

Thuong et al. [20] suggested that silica filler has a more prominent effect than styrene filler in their study. The rubber composite incorporated with both silica and styrene exhibit outstanding tensile strength than the samples reinforced with styrene alone. Although the inclusion of silica to rubber composites has the potential to increase mechanical properties, the amount employed is crucial. The tear strength of silica filled natural rubber composite significantly improved compared to natural rubber composite without added silica according to Dileep et al. [17]. Tear strength of all silica filled natural rubber composite increases up to 20 phr of silica loading and then decreases slightly. This phenomenon is explained by agglomeration of the silica particles at higher loading which results in poor dispersion and consequently lower mechanical properties. This trend is in agreement with the results obtained by Sadequl et al. [24]. In another research by Charoenchai et al. [18], the mechanical properties of neat natural rubber did not show significant change when compared to reinforced rubber composites due to low content of silica used in the system. Sombatsompop et al. [23] concluded that the tensile strength stabilized at 30 phr of silica content and further increment of silica content only increased the crosslink density of the vulcanizates without providing any mechanical enhancement. The decrease in mechanical properties was due to low interaction between filler and the rubber component. 

### 2.3. Lignocellulosic Fibre

Table 3 summarizes the main findings of rubber composite reinforced with lignocellulosic fiber filler from available literatures.

**Table 3:** The summary of literatures using lignocellulosic fiber as fillers for rubber composite.

Lignocellulosic Fiber/Filler	Treatments	Variables	Properties Tested and Findings	References
Torrefied almond shells (TAS) and torrefied rice hulls (TRH)	torrefaction	Carbon black to torrefied filler loadings ratio (40:20, 30:30, 20:40)	**Curing Characteristic** The curing time increased with higher loadings of both torrefied fillers. **Tensile Strength and Modulus** Generally, carbon black filled composite showed better mechanical properties than torrefied filler reinfored composite. The tensile strength of TAS filled natural rubber composite decreased with decreasing carbon black to torrefied filler ratio, while TRH filled natural rubber composite and reached the lowest tensile strength at 30:30 ratio before increasing significantly at 40:20 ratio. The modulus of TAS filled natural rubber composite showed similar trend with its tensile strength. On the other hand, the modulus of TRH rubber composite showed the lowest modulus at 40:20 ratio before increasing gradually at 30:30 and 20:40 ratio.	[25]
Horsetail (*Equisetum Arvense*)		Horsetail filler loading (10, 20, 30, 40 50 phr)	**Elongation at Break** The elongation at break increases with increasing horsetail loading from 10 phr to 50 phr. **Tensile Strength** Tensile strength of rubber composite reinforced with horsetail show higher value than the pure natural rubber sample. Initially, tensile strength increases with the addition of horsetail filler then drop slightly as the loading increases.	[26]
Cereal straw	Silane treatment	Silanes coupling agents (PTES, VTES, TESPTS), filler loading (10, 20, 30 phr)	**Elongation at Break** The elongation at break decreased with increasing cereal straw filler from 10 to 30 phr) **Tensile Strength** Rubber composites with silanes modified filler show improved tensile strength compared to composite with unmodified filler and the control specimens. The tensile strength increased and peaked at 10 phr loading and decreased with higher filler content.	[27]
Hemp fibre	Silane (Si69) and permanganate (KMnO_4_) treatment	Filler loading (5, 10, 15 phr)	**Curing Characteristic** Generally, the curing time of rubber composites increases with increasing hemp loading. The curing time for rubber composites filled with both silane or permanganate treated fibre is longer than the untreated counterparts. **Tensile Strength and Modulus** This finding shows increased tensile strength of silane treated fibre rubber composites compared with untreated and permanganate treated hemp fiber filled rubber composites. Tensile strength increased with filler loading and peaked at 10 phr before showing a decrease trend at 15 phr. The modulus however demonstrated an continual increase trend with higher filler loading.	[28]
Coconut shell powder	Alkali treatment	Filler loading (10, 20, 30, 40 phr)	**Curing Characteristic** Curing time was found to decrease consistently with increasing filler loading **Tensile Strength and Modulus** Tensile strength of the natural rubber composite was highest at 10 phr loading and decreased with increasing filler loading of 20 to 40 phr. Generally, the modulus showed similar trend with tensile strength.	[29]
Rice husk	Electron beam irradiation	Irradiation dosage	**Tensile Strength and Modulus** Max stress increases with irradiation dosage until about 20 kGy and decreases with further increase of radiation. However, the modulus seems to maximize at about 30 kGy of radiation.	[30]
Wood flour	Corona treatment in air and in ammonia	Filler loading (10, 20, 30, 40, 50, 60, 70 phr)	**Curing Characteristics** The higher the filler loading the longer the curing time. Nonetheless, the addition of treated wood flour treated with corona in air and ammonia did not significantly affect the vulcanization process of the rubber matrix. **Tensile Strength and Modulus** The tensile strength increased with filler laoding at 10 phr and decreased consistently with filler loading of 20, 30, 40 and 50 phr. However, the tensile strength increased again at 70 phr filler loading	[31]
Oil palm wood flour		Filler loading	**Tensile Strength and Modulus** The results show that increasing the concentration of oil palm wood flour increases the tensile modulus. However, the tensile strength and the elongation at break show a reverse trend.	[32]

According to Table 3, the curing characteristic and mechanical properties vary widely and are influenced by various variables or a combination of variables such as the type of lignocellulosic fiber filler, the treatment applied on the filler as well as the loadings of the filler used in natural rubber composites. Based on Table 3, it can be summarized that the curing time of natural rubber composite increased with increasing lignocellulosic fiber filler loading. Generally, natural rubber composite reinforced with 10 phr filler loading exhibited the strongest tensile properties. Further increment in filler loading showed a decline in tensile strength but increased the modulus of the rubber composite.

Torres et al. [25] and Moonart and Utara [28] concluded that the curing time increased consistently with increasing filler loading. The increase in curing time suggested that a retardation effect occurred during the curing process. They speculate that the accelerators involved in speeding up the curing process were trap in the porous structure of lignocellulosic filler, thus affected the curing characteristic of rubber composite. Sareena et al. [29] however reported a reverse trend. Vladkova et al. [31] reported that the addition of corona treated lignocellulosic filler did not significantly influence the curing time. However, increasing the filler loading did increase the curing time of natural rubber composite. The mechanisms of this phenomena are similar to those of carbon black and silica filler.

In 1998, Ismail and Nurdin [32] conducted research to determine the tensile properties of rubber composite reinforced with oil palm wood flour. They discovered that when the concentration of oil palm wood flour increased, the tensile modulus increased, however the tensile strength and elongation at break decreased. Although scanning electron microscopy has demonstrated an improvement in the surface interaction between oil palm wood flour and rubber components, systematic research on the influence of wood flour in rubber composites is lacking. According to Vladkova et al. [31], corona treatment under ideal operating circumstances can improve the efficiency of wood flour as a filler in natural rubber composites. Corona treatment in air, according to the authors, is a more effective method for increasing the wood flour reinforcing effect in nonpolar rubbers. Corona treatment in ammonia, on the other hand, causes a more complex change in the chemical composition of the wood surface due to the suppression of surface oxidation and the build-up of nitrogen-containing groups.

**Figure 3:**
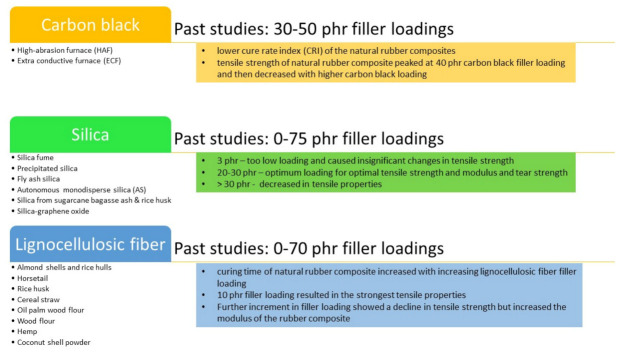
Summary of the effects of different types of filler and filler loadings on the properties of natural rubber composites.

Sareena et al. [29] studied the effect of coconut shell powder loadings on the mechanical properties of rubber composite. They noted that samples with filler loading at 10 phr exhibited highest tensile strength and showed highest modulus value as this loading concentration provides a large interfacial area of contact, resulting in better interfacial adhesion. Any higher filler loading will cause weak interaction and bonding between the filler particles and the natural rubber component which was responsible for the decline of tensile strength. This is in agreement with Miedzianowska et al. [27], which observed in their study that initial increase of cereal straw filler (10 phr) also increased the tensile strength but further increment (20 and 30 phr) reduced the tensile strength slightly. However, it is worth noting that the cereal filler used was treated with silanes coupling agents i.e., propyltriethoxysilane (PTES), vinyltriethoxysilane (VTES) and 3,3′-Tetrathiobis (propyl-triethoxysilane) (TESPTS). The TESPTS and VTES modified rubber composite exhibited the most significant changes in mechanical properties. Improvement in the mechanical properties of composites may result from increased adhesion of fibers to the polymer, and thus stronger interfacial interactions influencing the increase in cross-linking density. Torres et al. [25] studied the effects of carbon black to torrefied fillers ratio on the tensile strength and modulus of natural rubber composite. The authors observed that the introduction of higher torrefied fillers loading reduced the tensile strength and modulus of the hybrid natural rubber composite. They suggested that the residue of hydroxyl or carbonyl group after torrefaction treatment might reduce the interaction between rubber matrix and filler (almond shells or rice hulls). Additionally, the torrefied filler might be unable to support stresses transferred from the rubber matrix. This is in line with the findings by Masłowski et al. [26]. The authors also noted that for rubber composite filled with 10 phr of horsetail, the tensile strength increased by a significant 27%. Nevertheless, further addition of horsetail content triggered a slight decrease in the tensile strength. It is worth noting that, with 50 phr horsetail loading, the tensile strength was the lowest but still slightly higher than natural rubber composite without the addition of horsetail filler. Figure 3 summarizes the effects of different types of filler and filler loadings on the properties of natural rubber composites.

## 3. Types of Nanocellulose Fillers from Natural Fiber

As previously stated, carbon black is regarded as the most commercially suitable filler for natural rubber. However, in recent years, nanocellulose has demonstrated excellent potential in replacing carbon black. In fact, nanocellulose (NC), cellulose in the form of nanostructures, has been proven as one of the most promising sustainable materials of the future in recent decades. Kulshrestha et al. [33] demonstrated that the reinforcing ability of a hybrid filler system including 2 phr CNFs and 50 phr carbon black was practically equivalent to that of 65 phr carbon black in natural rubber compounds in terms of mechanical behavior. As a result, 2 phr CNFs might potentially replace roughly 15 phr carbon black in natural rubber formulations. Furthermore, when subjected to repeated loading cycles, natural rubber nanocomposite reinforced with CNC maintains stiffness better than neat natural rubber [34]. In their study, the stress softening effect of natural rubber nanocomposite reinforced with thiol-modified CNC was 4–6 times better than natural rubber reinforced with carbon black as reported by Harwood et al. [35]. There are many types of nanocellulose which can be generally classified into microfibrillated cellulose (MFC), cellulose nanocrystals (CNCs) and bacterial nanocellulose (BNC).

### 3.1. Microfibrillated Cellulose (MFC)

Generally, MFC is defined as cellulose nanomaterials consisting of fibrils and also known as cellulose nanofibrils (CNFs) and nanofibrillated cellulose (NFC) [36,37,38,39]. It has the same properties but different in size, with diameters of 5–30 nm and up to several microns long [40]. Muqeet et al. [41] stated the CNFs can be easily modified through substitution reaction because of their abundant hydroxyl groups. Besides that, cellulose-based nanomaterials have also been studied extensively as adsorbents, owing to their high surface area to volume ratio and the flexibility of functionalization in various fashions.

### 3.2. Cellulose Nanocrystals (CNCs)

CNCs, sometimes knows as nanocrystalline cellulose (NCC) and cellulose nanowhiskers (CNW), are widely used and preferable for industrial needs, with their characteristic of high mechanical strength plus flexibility of surface chemistry [42,43]. Under magnification, CNC represents a rod-shaped like crystalline form [44]. The main contribution in CNC properties is their cholesteric (Ch) liquid crystalline structures in liquid form. This ability helps the industry in terms of humidity sensors, optical encryptors, structural pigments, light shutters, and templates to synthesize inorganic materials. The CNC are becoming the industrial favorite component used due to their renewable characteristic and significant application as a reinforcing agent. Besides that, in the form of the solidified film, the CNC appears as a structural color that can be altered by additives (nanoparticles, organic dyes, and surfactants) [45]. Compared to CNFs, CNC has a stiffer structure due to higher crystallinity resulting from the removal of the amorphous region during the acid hydrolysis [46]. CNC has 3 to 5 nm of width and 100 nm of length up to several micrometers [46]. The CNC particles are highly crystalline materials varying from 54 to 88% in crystallinity and are 100% cellulose with a higher thermal stability value (~260 °C), larger aspect ratio (10 to 70), and lower density value (1.5–1.6 g/cm^3^) [46]. 

### 3.3. Bacterial Nanocellulose (BNC)

BNC is the extracellular product that produces by bacteria with high purity [47,48]. BNC has a diameter size of 20–100 nm and ribbon-like shape [40]. Gluconoacetobacter is an example of a bacteria type that BNC is extracted from, and it was grown in liquid culture media [46]. Carbon is used as the energy source for the bacteria culture and the nitrogen for the growing culture. BNC has almost similar properties to CNC and CNF, but more often, it is lightweight, non-toxic, and controls microfibril formation depending on the bacterial culture parameters [46]. The application of BNC is famous in biomedical applications such as wound healing and regenerative medicine. 

## 4. Surface Modification of Nanocellulose for Natural Rubber Nanocomposites

Chemical modification is often carried out to enhance the interfacial compatibilities between nanocellulose and natural rubber and to alleviate difficulties in dispersing them in polar solvents or polymers [49]. Hydroxyl groups are the sole functional group that exists in nanocellulose and therefore the surface modification of nanocellulose is highly dependent of the reactivity of these functional groups. In the hydrogen bonding-induced aggregation of materials, the nanoscale structure is critical. Under certain conditions, cellulose can be chemically changed in the presence of active sites in chains. The cellobiose ring is made up of three hydroxyl groups: secondary (C2 and C3) and primary (C6) alcohol groups, which allow it to be substituted for other functional groups and lengthy chains, and it may also be oxidized [50].

**Figure 4:**
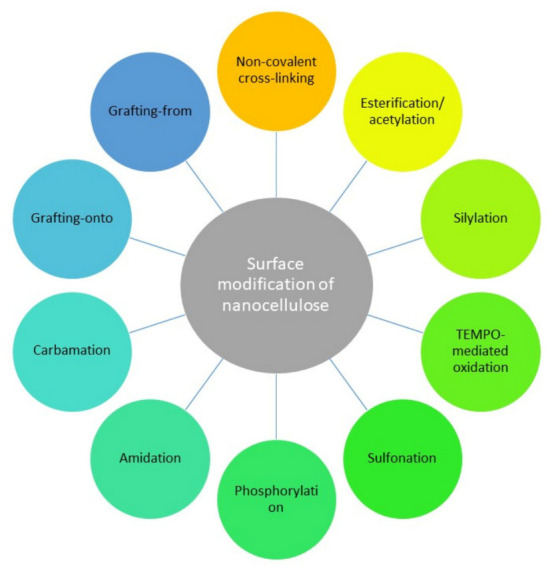
Surface modification methods of nanocellulose (summarized from Ghasemlou et al. [51]).

Figure 4 illustrates several surface modification methods of the nanocellulose which including esterification/acetylation, silylation, TEMPO-mediated oxidation, sulfonation, phosphorylation, amidation, carbamation, grafting-onto, grafting-from and non-covalent cross-linking. A comprehensive discussion on these methods could be found in the review compiled by Ghasemlou et al. [51].

Based on the available literatures, in the production of nanocellulose reinforced natural rubber nanocomposites, esterification, silylation and TEMPO-mediated oxidation are the most widely used surface modification methods to modify the nanocellulose. Table 4 summarizes the surface modification done on the nanocellulose and their reinforcement effect on the natural rubber nanocomposite.

### 4.1. Esterification

**Figure 5:**
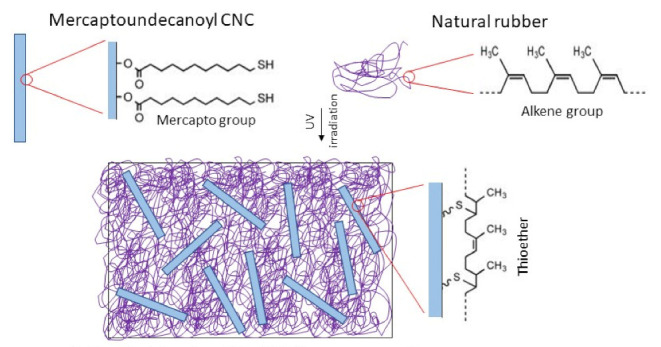
Illustration of the thiol-ene coupling reaction between natural rubber and modified cellulose nanocrystals in nanocomposite (adapted with permission from Kanoth et al. [34]. Copyright 2015 American Chemical Society, Washington, United States).

Esterification is the easiest reaction in order to remove the hydroxyl groups from nanocellulose. Esterification is a chemical reaction between acid (carboxylic acid) and alcohol (or other -OH) to form ester and water [62]. In a study by Kanoth et al. [34], cross-linkable mercapto group has been added covalently on the surface of cellulose nanocrystals (CNC) via esterification with 11-mercaptoundecanoic acid. The study intended to explore the synergistic effect of CNC as both reinforcing filler and crosslinker agent. The surface-grafted thiol (-SH) groups from 11-mercaptoundecanoic acid on CNC lead to thiol-ene coupling reaction with alkene groups in the molecules of natural rubber matrix to form covalent crosslinks (Figure 5). As a result, an effective chemical bonding between the CNC and natural rubber matrix interface is formed. Therefore, natural rubber nanocomposite reinforced with modified CNC has good dispersion and higher crosslink density than unmodified CNC. A uniaxial tensile test was conducted to evaluate the mechanical strength of the natural rubber/CNC nanocomposites. At similar nanocellulose content (10 wt%), natural rubber nanocomposite reinforced with modified CNC has significantly better tensile strength (10.2 MPa vs. 4.2 MPa), strain-to-failure (750% vs. 1210%), modulus (1.75 MPa vs. 1.86 MPa) and work-of-fracture (1.56 MJ m^−3^ vs. 4.60 MJ m^−3^) than that of natural rubber nanocomposite reinforced with unmodified CNC. In addition, natural rubber nanocomposite reinforced with modified CNC also has better preservation of stiffness than unmodified CNC nanocomposites when subjected to repeated loading cycles. The authors reported that the stress softening effect of natural rubber nanocomposite reinforced with thiol-modified CNC in their study was 4–6 times better than the natural rubber reinforced with carbon black as reported by Harwood et al. [35].

Apart from that, esterification of nanocellulose could also be carried out using unsaturated fatty acids such as oleic acid and stearic acid. Natural rubber reinforced with oleic acid and stearic acid modified cellulose nanofibers (CNF) at CNF content of 5 wt% portrayed a transparent feature, indicates well dispersion of modified CNF in natural rubber [59]. Esterification has enhanced the reinforcing efficiency of the CHF as natural rubber reinforced with modified CNF displayed better Young’s modulus than unmodified CNF at any level of CNF content (1, 3 and 5 wt%). It is noteworthy that oleoyl CNF with double bonds resulted in better mechanical behavior of the natural rubber composite compared to that of stearoyl CNF. Lower swelling of 241.1% for the oleoyl CNF/natural rubber nanocomposite was also observed compared to 272% in stearoyl CNF/natural nanocomposite. It can be concluded that oleoyl CNF with double bonds achieves higher level of interaction with natural rubber.

### 4.2. Silylation

**Figure 6:**
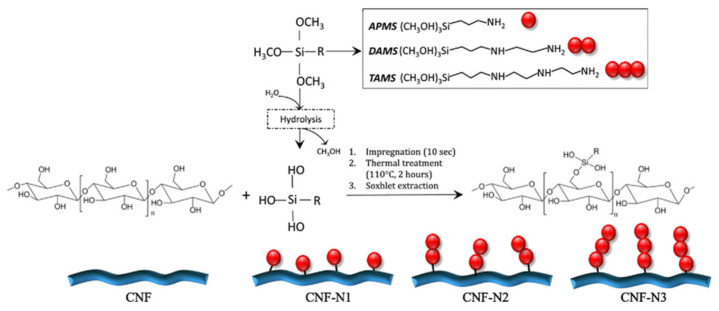
Schematic illustration of the mechanism for aqueous-based silylation reaction with amino silanes on the surface of cellulose nanofibers films (Saini et al. [63]; with permission from Elsevier Science Ltd., Amsterdam, Netherlands).

Silyation is one of the frequently used modification methods to surface modified nanocellulose where silyl groups are introduced to the surface of nanocellulose. Figure 6 displays the grating mechanism of different amino silanes on the hydroxyl groups of cellulose of cellulose nanofiber (CNF) films. Grafting of amino silanes happened on the accessible amorphous regions of CNF. Therefore, fibers thickening and formation of three dimension al silane networks at the surface of CNF films could be observed as result of self-condensation reaction [63].

Xu et al. [52] modified nanocrystalline cellulose (NCC) with 3-aminopropyl-triethoxysilane and the modified NCC were reinforced into natural rubber matrix with different NCC:silica ratio. Addition of both unmodified NCC and modified NCC accelerated the vulcanization rate of natural rubber. The Payne effect, a particular feature of the stress-strain behavior of rubber, decreased along with increasing content of unmodified and modified NCC. Meanwhile, the mechanical properties of the natural rubber nanocomposites were significantly improved by the addition of modified NCC compared to that of unmodified NCC as a result of a more uniform dispersion of modified NCC in the natural rubber matrix. By the means of scanning electron microscopy (SEM) micrographs, it was revealed that the unmodified NCC aggregated more heavily in the natural rubber matrix. On the contrary, more uniform dispersion and lesser aggregations were found in the case of modified NCC, resulted in better bonding between natural rubber and nanocellulose. Consequently, nanocomposites reinforced with modified NCC portrayed better performance on the macroscale.

**Figure 7:**
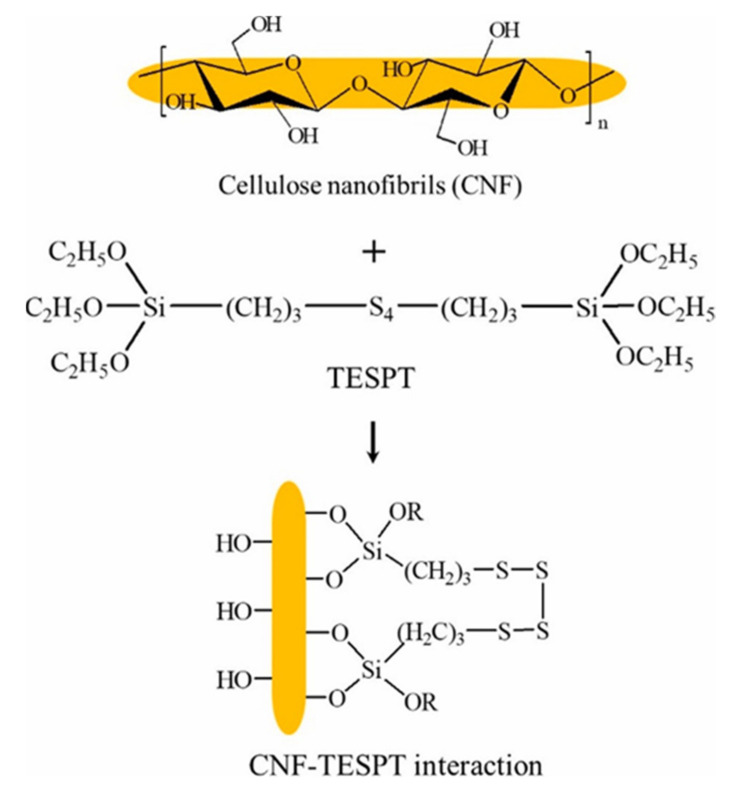
Proposed mechanism of interaction between CNF and TESPT (Somseemee et al. [55]; with permission from Elsevier Science Ltd., Amsterdam, Netherlands).

The crystallinity of the nanocellulose was reduced by chemical modification and subsequently weakened its reinforcement potential in polymer composites [64]. However, Singh et al. [54] proved that the silylation treatment did not affect the crystallinity of the CNC adversely as the modification only occurred mostly at the surface of the CNC. In a study by Somseemee et al. [55], the crystallinity of the CNF modified by Bis-(triethoxysilyl-propyl) tetrasulfide (TESPT) showed a slight decrease from 74.3% to 69.5%. Similar to the previous studies, better dispersion is observed for modified CNC in the natural rubber matrix. Unmodified CNC exhibited agglomerated nature owing to the internal hydrogen binding between the spherical CNC particles. In addition, non-polar natural rubber also restricted the well-dispersion of polar CNC in the natural rubber matrix. This problem was overcome by the surface modification of CNC with (3-Aminopropyl)triethoxysilane (APTES), where the polar nature of the CNC was reduced after modification [54]. Moreover, aliphatic chains from APTES could coat over the surface of CNC and improve its compatibility with natural rubber. The chemical reaction between the hydroxyl group of nanocellulose and hydrolyzable alkoxy group of TESPT has contributed to the improvement in compatibility of nanocellulose with natural rubber (Figure 7).

Nevertheless, some contrary findings have also been reported. Kargarzadeh et al. [53] modified cellulose nanocrystals (CNCs) using 0.5 wt% silane. The modified CNC was then used to prepared unsaturated polyester resin (UPR) nanocomposites toughened with liquid natural rubber (LNR). The study revealed that the reinforcement with both unmodified and modified CNC improved the tensile properties, impact energy, viscoelastic behavior and thermal resistance of the nanocomposite. However, when comparing between unmodified and modified CNC, nanocomposites reinforced with silane modified CNC displayed lower tensile strength and modulus compared to that of its unmodified CNC counterparts. The authors attributed the decrement in tensile properties to the weaker intramolecular interactions between the hydroxyl groups of the modified CNC with the LNR. Therefore, a CNC network failed to form and the stress transferring between rubber and UPR was lessened which subsequently led to inferior tensile strength. In addition, the fact that the crystallinity of CNC was reduced by the silane treatment and its subsequent lesser stiffening effects has also contributed to the decrement in tensile strength of the nanocomposites [65]. Additionally, the lesser crystalline and rigid characteristics of silane modified CNC also resulted in lower glass transition temperature (T_g_) compared to that of unmodified CNC. Nevertheless, higher impact properties were observed from the nanocomposites reinforced with modified CNC, mainly due to their lower propensity toward self-aggregation. Correspondingly, nanocomposites with a more flexible filler-matrix interface and reduced stress concentrations could be fabricated. Based on the findings, it was concluded that the use of CNC modified with silane is less preferential as the treatment weakens the interaction between CNC and LNR.

### 4.3. TEMPO-Mediated Oxidation

**Figure 8:**
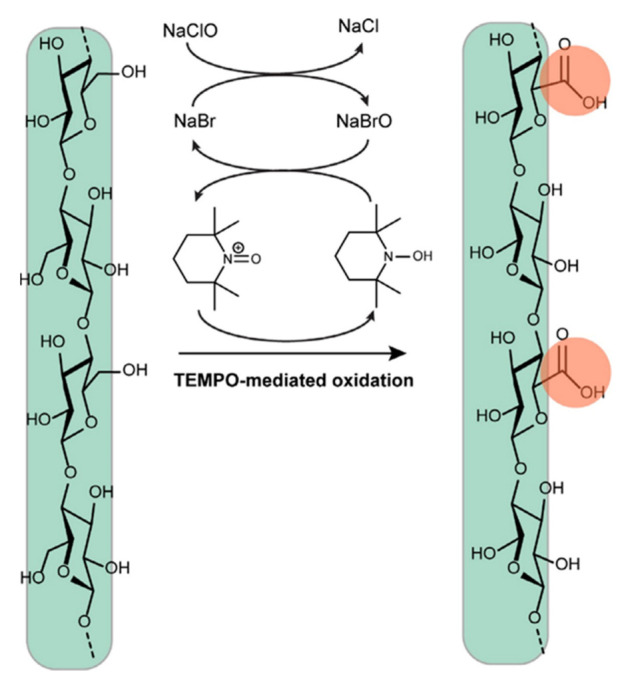
Schematic illustration of the TEMPO-mediated oxidation where it selectively transforms C6-hydroxy groups of nanocellulose to carboxylate groups (Ghasemlou et al. 2021; with permission from Elsevier Science Ltd., Amsterdam, Netherlands).

According to Pierre et al. [66], TEMPO-mediated oxidation is deemed as an effective treatment to enhance the stability of nanocellulose suspensions via bestowing negative charges onto the surface of nanocellulose. As shown in Figure 8, during the TEMPO-mediated oxidation, the C6 hydroxy groups of nanocellulose transform into carboxylate groups selectively, in the presence of sodium hypochlorite (NaClO) and sodium bromide (NaBr) [51]. These carboxylate groups could then assist in obtaining nanocellulose with different functionalities by acting as a platform to gather metals ions by ion exchange [67].

Mahendra et al. [56] TEMPO-oxidized nanocrystalline cellulose (NCC) and nanofiber cellulose (NFC) from lower part of empty fruit bunches and reinforced them into polypropylene (PP)/cyclic natural rubber (CNR) (80/20) nanocomposites. NCC produced in this study has larger diameter and lower aspect ratio (L/D) compared to that of the NFC. Consequently, owing to its larger dimensions, NCC has higher degradation temperature than NFC. Generally, PP/CNR nanocomposites exhibited inferior tensile strength when modified NCC and NFC was added and the decrement in tensile strength increased with increasing nanocellulose content from 1 to 3 wt%. The reduction in tensile strength is a common observation in natural rubber composites reinforced with lignocellulosic fibers. The fibers are more rigid and therefore will experience fractures earlier than the natural rubber matrix. Similar observation was found for Young’s modulus in the PP/NCR nanocomposite reinforced with NCC. However, Young’s modulus of the PP/NCR nanocomposite reinforced with NCC reinforced with NFC was higher and increased along with increasing nanocellulose content. As NFC has higher aspect ratio than NCC, the interfacial contact surface of NFC and CNR are better and hence enhanced reinforcement effect was obtained. SEM micrographs also proved that NFC resulted in a better dispersion in CNR matrix compared to that of NCC. On the contrary, NCC reinforced PP/CNR nanocomposite possesses higher thermal stability than that of its NFC counterpart.

### 4.4. Other Modifications

In a study by Jiang et al. [60], nanocrystalline cellulose (NCC) modified by cetyltrimethyl ammonium bromide (CTMAB) has been reinforced into natural rubber at an NCC content of 0, 5, 10, 15 and 20 wt%. Modification with CTMAB has increased the size of the NCC as CTMAB was absorbed on the surface of NCC. Additionally, the charge on the surface of NCC became positive after modification. In comparison to unmodified NCC, CTMAB modified NCC exhibited slightly lower crystallinity index. However, the crystallinity of NCC is still considered high after modification. The authors also reported that the modified NCC may have assisted in accelerating the vulcanization of natural rubber and shorter curing time was observed in modified NCC nanocomposites. One of the prominent observations is the improvement of dispersion of NCC in the natural rubber as the surfactant on the NCC surface prevented the agglomeration of NCC. Nevertheless, it only could be observed when low NCC content (5 and 10 wt%) was used. Modification has improved the interfacial interaction between NCC and natural rubber as proved by the decrement of tan δ in glass transition region. Similar findings were also recorded for mechanical properties of the nanocomposites. At 5 and 10 wt% NCC content, natural rubber nanocomposites reinforced with modified NCC displayed better tensile strength, tear strength and abrasion resistance compared to that of the unmodified NCC. The improvement in mechanical properties is mainly attributed to improved dispersion and enhanced interfacial interaction as a result of surface medication of NCC. When the NCC content was 15% and above, the dispersion reduced and resulted in inferior mechanical properties. Moreover, addition of modified NCC also resulted in better wet-skid resistance and aging resistance of the resultant nanocomposites. Overall, addition of 10% CTMAB modified NCC led to the optimum properties.

Apart from the aforementioned methods, the surface of nanocellulose could also be decorated with polystyrene. A study by Trovatti et al. [61] demonstrated admicelar polymerization of polystyrene at the surface of the bacterial cellulose (BC) nanofibers. The purpose of the treatment was to ensure a better dispersion into the natural rubber matrix by reducing its hydrophilicity and polarity. The polystyrene coated BC nanofibers remained its original morphology but with more hydrophobic nature and decreased surface energy as a result of partially masking of hydroxyl groups by the polystyrene sleeve. Tensile strength and Young’s modulus of the nanocomposites reinforced with unmodified BC nanofibers and polystyrene coated BC nanofibers exhibited significant improvement. However, no significant difference in terms of mechanical performance between the two. It should be noted that, at lower BC nanofibers content (1 and 2.5 wt%), the resultant nanocomposite did not differ much with the neat natural rubber. The advantages of the incorporation of BC nanofibers could only be shown when higher content (5 and 10 wt%) of BC nanofibers were added to the natural rubber matrix.

## 5. Conclusions, Challenges and Future Perspectives

As the demand from automotive industry keep increasing over the years, the application and supply of natural rubber is undoubtedly pivotal. However, environmental issues and health hazards that triggered by the rubber industry is a problem that has come to the foreground in recent years. More and more stringent environmental regulations have been imposed to prevent the rubber industry to produce some types of rubber. For instance, butyl rubber has been classified as a major source of Hazardous Air Pollutants (HAP) emission by The Environmental Protection Agency (EPA) during its production process. Prolonged and acute exposure to the chemicals used in the production of industrial rubber could be detrimental and lethal to humans’ health. Moreover, the majority of the raw materials used are petroleum-based and non-renewable and therefore lead to high environmental impact [68].

Therefore, the development of green-based natural rubber materials has become more and more important in ensuring environmental safety. The demand for eco-friendly rubber is increasing worldwide. At this moment, the advantages of nanocellulose derived from renewable resources as reinforcing filler to natural rubber has become irreplaceable. However, the dispersion of nanocellulose into the natural rubber matrix is one of the main challenges during the production of nanocellulose reinforced nanocomposites [69]. Another problem in the production of these nanocomposites is the poor compatibility of hydrophobic natural rubber and hydrophilic nanocellulose [70]. Various surface modification methods of nanocellulose have been reported in this review. Encouraging breakthrough and improved nanocellulose dispersion into the natural rubber matrix via esterification, silylation and TEMPO-mediated oxidization has been reported. Better interfacial compatibility was achieved and subsequently nanocomposites with improved mechanical, curing, dynamic mechanical and thermal properties were produced.

Even so, the commercial suitability and the ability of large-scale processing of these nanocomposites is still questionable [71]. Apart from that, the cumbersome process also makes the production of nanocellulose intensively laborious. Consequently, manufacturers are always facing dilemma in judging and weighing between the advantages bestowed by nanocellulose and the disadvantages of arduous processing of nanocellulose reinforced natural rubber nanocomposites. Volatile global rubber price is also an issue that must be paid attention to as the price of rubber is anticipated to slowly rise up to the year 2030.

Despite the aforementioned challenges, nanocellulose based natural rubber composites are still considered as very promising future materials to be used in many different sectors. The evidence from several past studies in this review suggests that the natural rubber nanocomposites reinforced with surface modified nanocellulose could be potentially used in elastic packaging in food and medical applications owing to their excellent mechanical properties and thermal stability. Meanwhile, thanks to the environmentally friendly nature and biodegradability of nanocellulose, plenty of green composites with superior thermal, mechanical and barrier properties could be produced. Nanocellulose could serve as an ideal filler in the automobile industry in producing green tires [72].

## Figures and Tables

**Table 1:** The summary of literature using carbon black as the main filler for natural rubber composite.

Filler/Source	Treatments	Variables	Properties Tested and Findings	References
Carbon black (N220), silica	-	Carbon black to silica ratio (30:15, 40:15, 50:15)	**Tensile and Tear Strength** Generally, tensile and tear strength increased as the carbon black to silica ratio increased and peaked at 40 phr carbon black content before decreased slightly. **Abrasion Resistance and Modulus** The composite with the highest carbon black content (50 phr) exhibited the highest abrasion resistant index (ARI) and modulus. The ARI and modulus increased proportionally to the carbon black content.	[13]
Carbon black	-	Type of carbon black (HAF and ECF)	**Curing Characteristic** The cure rate index (CRI) for rubber composite added with both type of carbon black showed significant lower value than rubber composite without reinforcement. **Tensile Strength** Both types of carbon black filler (HAF and ECF) decreased the tensile strength of natural rubber composite.	[14]
Carbon black	N-tert-butyl-2-benzothiazole sulfenamide (TBBS)	TTBS concentration (1.0, 1.4, 1.8, 2.2, 2.6 phr)	**Curing Characteristic** The cure rate index (CRI) was similar between carbon black (40 phr) and silica (3.2 phr) filled rubber composite. The CRI decreased with increasing TTBS concentration. **Tensile Strength and Modulus** Tensile strength and modulus of carbon black filled rubber composite (40 phr) showed higher tensile strength than silica reinforced rubber composite (3.2 phr) for all TTBS concentration. The tensile strength for carbon black filled rubber composite peaked at 2.0 phr TTBS concentration then decreased with higher concentration, while the modulus value continued to increase with increasing concentration.	[15]

**Table 4:** Recent reports on surface modified nanocellulose (NC) reinforced natural rubber nanocomposites.

Nanocellulose	Surface Modification	Nanocomposite	Findings	References
Nanocrystalline cellulose (NCC) from commercial microcrystalline cellulose (MCC)	Silylation—3-aminopropyl-triethoxysilane	Natural rubber (NR)/NCC/silica nanocomposite at different ratios of NCC and silica (0:30, 5:25, 10:20, 15:15, 20:10 and 25:5)	- Enhanced dispersion and interfacial strength for modified NCC - Nanocomposites reinforced with modified NCC exhibited higher tensile strength, modulus, elongation at break, tear strength and hardness compared to that of unmodified NCC	[52]
Cellulose nanocrystals (CNC) from kenaf bast fiber	Silylation—0.5 wt% silane	CNC reinforced unsaturated polyester resins (UPR) toughened with liquid natural rubber (LNR) at 2, 4 and 6 wt% CNC content	- Compatibility between UPR and LNR has been improved after reinforced with modified CNC - Lower tensile strength and modulus but higher impact energy was observed for modified CNC compared to that of unmodified CNC nanocomposite - Viscoelastic behavior and thermal resistance of the modified CNC nanocomposite was slightly lower than unmodified CNC	[53]
Crystalline nanocellulose (CNC) from ramie fiber	Silylation—Different organosilanes: (3-Aminopropyl)triethoxysilane (APTES), 3-aminopropyl-triethoxysilane, bis[3-(triethoxysilyl)propyl] tetrasulfide (TESPT), (3-mercaptopropyl) trimethoxysilane (MPTMS)	Natural rubber (NR)/CNC nanocomposite at 2.5 and 5 wt% CNC content	- Crystallinity index of unmodified CNC, APTES-MCNC, TESPT-MCNC, and MPTMS-MCNC was 74%, 66%, 70% and 51%, respectively. - Tensile properties of the natural rubber reinforced with modified CNC was significantly higher than that of unmodified CNC	[54]
Cellulose nanofibrils (CNF) from Napier grass stem	Silylation—Bis-(triethoxysilyl-propyl) tetrasulfide (TESPT)	Natural rubber (NR)/CNF nanocomposite at 0.5, 1, 3, 5 and 10 wt% filler loading	- Lower degree of crystallinity of CNF after modified with TESPT (74.3% in unmodified CNF and 69.5% in modified CNF) - TESPT-modified CNF has lower onset temperature and maximum decomposition temperature compared to unmodified CNF - Better rubber-filler interaction (indicates by bound rubber content) was observed in modified CNF filled nanocomposite - Greater reinforcing effect was shown by modified CNF as higher modulus and hardness and tensile strength of the resultant nanocomposites was recorded - Performance of nanocomposite improved with increasing filler loading up to 5 wt% and level-off beyond this loading level	[55]
Nanocrystalline cellulose (NCC) and nanofiber cellulose (NFC) from lower part of empty fruit bunches	TEMPO-mediated oxidation—2,2,6,6-tetramethyl-1-piperidinyloxyl	Polypropylene (PP)/cyclic natural rubber (CNR)/NCC or NFC nanocomposite with 1, 2 and 3 wt% NCC content	- Addition of NCC decreases the tensile strength and modulus of the PP/CNR nanocomposites by 13 and 56%, respectively and 56% higher in elongation at break was recorded - 16 and 25% increment in tensile strength and modulus was recorded when NFC was added and 5% decrement in elongation at break was observed - Nanocomposite added with NCC has better thermal stability than NFC	[56]
Commercial cellulose nanofibers (CNF)	TEMPO-mediated oxidation—2,2,6,6-tegramethylpyperidine-1-oxyl	TEMPO-CNF/ nitrile-butadiene rubber (NBR) and sheets and carboxy group-containing nitrile-butadiene rubber (XNBR) composite	- Tensile strength, storage modulus at 23 °C, work of fracture, and elongation at break of the TEMPO-CNF/XNBR nanocomposites are higher than that of control XNBR nanocomposites	[57]
Nanofibrillated cellulose (NFC) from bleached softwood bisulfite pulp	TEMPO-mediated oxidation—2,2,6,6-tetramethylpiperidine-1-oxyl	NFC reinforced latex nanocomposite at 1, 2, 3, 4 and 5 wt% NFC content	- The ultimate strength and elastic modulus of the nanocomposites improved significantly with addition of up to 3 wt% NFC content while 5 wt% resulted in the highest values	[58]
Cellulose Nanocrystals (CNC) from cotton cellulose	Esterification of thiols—mercaptoundecanoic acid mixed with acetic anhydride, glacial acetic acid, and concentrated sulfuric acid	Natural rubber/CNC nanocomposite at 2, 5 and 10 wt% CNC content	- Smooth and homogenous surface structure similar to that of the neat natural rubber was observed after incorporation of modified CNC - Higher crosslink density and significantly increased tensile strength and strain-to-failure observed in modified CNC nanocomposite compared to unmodified CNC - The improvement was significant at 5 wt% CNC content but level-off when 10 wt% CNC content was used	[34]
Cellulose nanofibers (CNF) from never-dried bleached softwood kraft pulp	Esterification—unsaturated fatty acids (oleic acid and stearic acid)	sulfur-vulcanized natural rubber reinforced with CNF at 1, 3 and 5 wt%	- Good dispersion of modified CNF was observed as the resultant natural rubber was transparent at CNF content of 5 wt% - 2 to 3 fold increment in Young’s modulus when 1 and 3 wt% modified CNF was added to the natural rubber compared to unmodified CNF. At higher CNF content (5 wt%), the increment is more significant - Oleoyl CNF displayed higher interaction with natural rubber compared to stearoyl CNF	[59]
Nanocrystalline cellulose (NCC) from softwood pulp	Non-covalent surface modification—cationic surfactant, cetyltrimethyl ammonium bromide (CTMAB)	Natural rubber (NR) composite reinforced with NCC at 5, 10, 15 and 20 wt%	- Lower crystallinity was observed in modified NCC (69.4%) compared to unmodified NCC (72.2%) - Better dispersion in NR was observed for modified NCC - Modified NCC accelerated the vulcanization process of NR - At lower NCC content (≤10 wt%), NR composites reinforced with modified NCC displayed superior tensile strength, tear strength and abrasion resistance than their unmodified NCC counterparts	[60]
Bacterial cellulose (BC) from modified Hestrin Shran liquid culture medium	Admicelar polymerization of styrene at the surface of the BC nanofibers	Natural-rubber based nanocomposites reinforced with bacterial cellulose (BC) and bacterial cellulose coated with polystyrene (BCPS) at 1, 2.5, 5 and 10 wt%	- Nanocomposites with low BC and BCPS fibers content (1 and 2.5 wt%) did not show significant improvement compared to natural rubber - At higher fiber content (5 and 10 wt%), the tensile strength and Young’s modulus of the nanocomposites increased significantly - No significant difference was found between BC and BCPS	[61]

## Data Availability

Not applicable.

## References

[1] Van Beilen J.B., Poirier Y. (2007). Establishment of new crops for the production of natural rubber. Trends Biotechnol..

[2] Cornish K. (2001). Similarities and differences in rubber biochemistry among plant species. Phytochemistry.

[3] Cataldo F. (2000). Guayule rubber: A new possible world scenario for the production of natural rubber. Prog. Rubber Plast. Technol..

[4] Wikipedia Natural Rubber. https://en.wikipedia.org/wiki/Natural_rubber.

[5] Statista Leading Natural Rubber Producing Countries Worldwide in 2019 and 2020. https://www.statista.com/statistics/275397/caoutchouc-production-in-leading-countries/.

[6] Mordor Intelligence Natural Rubber Market—Growth, Trends, COVID-19 Impact, and Forecasts (2021–2026). https://www.mordorintelligence.com/industry-reports/natural-rubber-market.

[7] Expert Market Research Global Natural Rubber Market Outlook. https://www.expertmarketresearch.com/reports/natural-rubber-market.

[8] Mariano M., El Kissi N., Dufresne A. (2016). Cellulose nanocrystal reinforced oxidized natural rubber nanocomposites. Carbohydr. Polym..

[9] Zhou Y., Fan M., Chen L., Zhuang J. (2015). Lignocellulosic fibre mediated rubber composites: An overview. Compos. Part B Eng..

[10] Low D.Y.S., Supramaniam J., Soottitantawat A., Charinpanitkul T., Tanthapanichakoon W., Tan K.W., Tang S.Y. (2021). Recent developments in nanocellulose-reinforced rubber matrix composites: A review. Polymers.

[11] Kargarzadeh H., Mariano M., Huang J., Lin N., Ahmad I., Dufresne A., Thomas S. (2017). Recent developments on nanocellulose reinforced polymer nanocomposites: A review. Polymer.

[12] Nunes R.C., Thomas S., Maria H.J. (2017). Rubber Nanocomposites with Nanocellulose. Progress in Rubber Nanocomposites.

[13] Sivaselvi K., Varma V.S., Harikumar A., Jayaprakash A., Sankar S., Krishna C.Y., Gopal K. (2021). Improving the mechanical properties of natural rubber composite with carbon black (N220) as filler. Mater. Today Proc..

[14] Salaeh S., Nakason C. (2012). Influence of modified natural rubber and structure of carbon black on properties of natural rubber compounds. Polym. Compos..

[15] Choi S.S., Nah C., Jo B.W. (2003). Properties of natural rubber composites reinforced with silica or carbon black: Influence of cure accelerator content and filler dispersion. Polym. Int..

[16] Abdul Wahab M.K., Ismail H., Othman N. (2012). Effects of dynamic vulcanization on the physical, mechanical, and morphological properties of high-density polyethylene/(natural rubber)/(thermoplastic tapioca starch) blends. J. Vinyl Addit. Technol..

[17] Dileep P., Varghese G.A., Sivakumar S., Narayanankutty S.K. (2020). An innovative approach to utilize waste silica fume from zirconia industry to prepare high performance natural rubber composites for multi-functional applications. Polym. Test..

[18] Charoenchai M., Tangbunsuk S., Keawwattana W. (2020). Silica-graphene oxide nanohybrids as reinforcing filler for natural rubber. J. Polym. Res..

[19] Boonmee A., Jarukumjorn K. (2020). Preparation and characterization of silica nanoparticles from sugarcane bagasse ash for using as a filler in natural rubber composites. Polym. Bull..

[20] Thuong N.T., Dung T.A., Yusof N.H., Kawahara S. (2020). Controlling the size of silica nanoparticles in filler nanomatrix structure of natural rubber. Polymer.

[21] Xia L., Song J., Wang H., Kan Z. (2019). Silica nanoparticles reinforced natural rubber latex composites: The effects of silica dimension and polydispersity on performance. J. Appl. Polym. Sci..

[22] Ahmed K., Nizami S.S., Riza N.Z. (2014). Reinforcement of natural rubber hybrid composites based on marble sludge/Silica and marble sludge/rice husk derived silica. J. Adv. Res..

[23] Sombatsompop N., Thongsang S., Markpin T., Wimolmala E. (2004). Fly ash particles and precipitated silica as fillers in rubbers. I. Untreated fillers in natural rubber and styrene–butadiene rubber compounds. J. Appl. Polym. Sci..

[24] Sadequl A.M., Poh B.T., Ishiaku U.S. (1999). Effect of filler loading on the mechanical properties of epoxidized natural rubber (ENR 25) compared with natural rubber (SMR L). Int. J. Polym. Mater..

[25] Torres L.F., McCaffrey Z., Washington W., Williams T.G., Wood D.F., Orts W.J., McMahan C.M. (2021). Torrefied agro-industrial residue as filler in natural rubber compounds. J. Appl. Polym. Sci..

[26] Masłowski M., Miedzianowska J., Czylkowska A., Strzelec K. (2020). Horsetail (*Equisetum arvense*) as a functional filler for natural rubber biocomposites. Materials.

[27] Miedzianowska J., Masłowski M., Rybiński P., Strzelec K. (2020). Properties of chemically modified (selected silanes) lignocellulosic filler and its application in natural rubber biocomposites. Materials.

[28] Moonart U., Utara S. (2019). Effect of surface treatments and filler loading on the properties of hemp fiber/natural rubber composites. Cellulose.

[29] Sareena C., Ramesan M.T., Purushothaman E. (2012). Utilization of coconut shell powder as a novel filler in natural rubber. J. Reinf. Plast. Compos..

[30] Chong E.L., Ahmad I., Dahlan H.M., Abdullah I. (2010). Reinforcement of natural rubber/high density polyethylene blends with electron beam irradiated liquid natural rubber-coated rice husk. Radiat. Phys. Chem..

[31] Vladkova T.G., Dineff P.D., Gospodinova D.N., Avramova I. (2006). Wood flour: New filler for the rubber processing industry. IV. Cure characteristics and mechanical properties of natural rubber compounds filled by non-modified or corona treated wood flour. J. Appl. Polym. Sci..

[32] Ismail H., Nurdin H.I. (1998). Tensile properties and scanning electron microscopy examination of the fracture surface of oil palm wood flour/natural rubber composites. Iran. Polym. J..

[33] Kulshrestha U., Gupta T., Kumawat P., Jaiswal H., Ghosh S.B., Sharma N.N. (2020). Cellulose nanofibre enabled natural rubber composites: Microstructure, curing behaviour and dynamic mechanical properties. Polym. Test..

[34] Kanoth P.B., Claudino M., Johansson M., Berglund L.A., Zhou Q. (2015). Biocomposites from natural rubber: Synergistic effects of functionalized cellulose nanocrystals as both reinforcing and cross-linking agents via free-radical thiol–ene chemistry. ACS Appl. Mater. Interfaces.

[35] Harwood J.A.C., Mullins L., Payne A.R. (1965). Stress softening in natural rubber vulcanizates. Part ii. Stress softening effects in pure gum and filler loaded rubbers. J. Appl. Polym. Sci..

[36] Zinge C., Kandasubramanian B. (2020). Nanocellulose based biodegradable polymers. Eur. Polym. J..

[37] Zhang K., Barhoum A., Xiaoqing C., Li H., Samyn P., Barhoum A., Bechelany M., Makhlouf A.S.H. (2019). Cellulose Nanofibers: Fabrication and Surface Functionalization Techniques. Handbook of Nanofibers.

[38] Gopakumar D.A., Thomas S., Grohens Y., Puglia D., Fortunati E., Kenny J.M. (2016). Nanocelluloses as Innovative Polymers for Membrane Applications. Multifunctional Polymeric Nanocomposites Based on Cellulosic Reinforcements.

[39] Supramaniam J., Wong S.K., Leo B.F., Tan L.T.H., Goh B.H., Tang S.Y. (2020). Unravelling the swelling behaviour and antibacterial activity of palm cellulose nanofiber-based metallic nanocomposites. IOP Conf. Ser. Mater. Sci. Eng..

[40] Mokhena T.C., John M.J. (2019). Cellulose nanomaterials: New generation materials for solving global issues. Cellulose.

[41] Muqeet M., Mahar R.B., Gadhi T.A., Halima N.B. (2020). Insight into cellulose-based-nanomaterials—A pursuit of environmental remedies. Int. J. Biol. Macromol..

[42] George J., Sabapathi S.N. (2015). Cellulose nanocrystals: Synthesis, functional properties, and applications. Nanotechnol. Sci. Appl..

[43] Abitbol T., Rivkin A., Cao Y., Nevo Y., Abraham E., Ben-Shalom T., Lapidot S., Shoseyov O. (2016). Nanocellulose, a tiny fiber with huge applications. Curr. Opin. Biotechnol..

[44] Vollick B., Kuo P.Y., Alizadehgiashi M., Yan N., Kumacheva E. (2017). From structure to properties of composite films derived from cellulose nanocrystals. ACS Omega.

[45] Leng J., Li G., Ji X., Yuan Z., Fu Y., Li H., Qin M., Moehwald H. (2018). Flexible latex photonic films with tunable structural colors templated by cellulose nanocrystals. J. Mater. Chem. C.

[46] Farooq A., Patoary M.K., Zhang M., Mussana H., Li M., Naeem M.A., Mushtaq M., Farooq A., Liu L. (2020). Cellulose from sources to nanocellulose and an overview of synthesis and properties of nanocellulose/zinc oxide nanocomposite materials. Int. J. Biol. Macromol..

[47] Stanisławska A. (2016). Bacterial Nanocellulose as a Microbiological Derived Nanomaterial. Adv. Mater. Sci..

[48] Klemm D., Cranston E.D., Fischer D., Gama M., Kedzior S.A., Kralisch D., Kramer F., Kondo T., Lindström T., Nietzsche S. (2018). Nanocellulose as a natural source for groundbreaking applications in materials science: Today’s state. Mater. Today.

[49] Gelir A., Yargi O., Yuksel S.A. (2017). Elucidation of the pore size and temperature dependence of the oxygen diffusion into porous silicon. Thin Solid Films.

[50] Kargarzadeh H., Mariano M., Gopakumar D., Ahmad I., Thomas S., Dufresne A., Huang J., Lin N. (2018). Advances in cellulose nanomaterials. Cellulose.

[51] Ghasemlou M., Daver F., Ivanova E.P., Habibi Y., Adhikari B. (2021). Surface modifications of nanocellulose: From synthesis to high-performance nanocomposites. Prog. Polym. Sci..

[52] Xu S.H., Gu J., Luo Y.F., Jia D.M. (2012). Effects of partial replacement of silica with surface modified nanocrystalline cellulose on properties of natural rubber nanocomposites. Express Polym. Lett..

[53] Kargarzadeh H., Sheltami R.M., Ahmad I., Abdullah I., Dufresne A. (2015). Cellulose nanocrystal reinforced liquid natural rubber toughened unsaturated polyester: Effects of filler content and surface treatment on its morphological, thermal, mechanical, and viscoelastic properties. Polymer.

[54] Singh S., Dhakar G.L., Kapgate B.P., Maji P.K., Verma C., Chhajed M., Rajkumar K., Das C. (2020). Synthesis and chemical modification of crystalline nanocellulose to reinforce natural rubber composites. Polym. Adv. Technol..

[55] Somseemee O., Sae-Oui P., Siriwong C. (2021). Reinforcement of surface-modified cellulose nanofibrils extracted from Napier grass stem in natural rubber composites. Ind. Crops Prod..

[56] Mahendra I.P., Wirjosentono B., Tamrin T., Ismail H., Mendez J.A., Causin V. (2020). The effect of nanocrystalline cellulose and TEMPO-oxidized nanocellulose on the compatibility of polypropylene/cyclic natural rubber blends. J. Thermoplast. Compos. Mater..

[57] Noguchi T., Niihara K.I., Kurashima A., Iwamoto R., Miura T., Koyama A., Endo M., Marubayashi H., Kumagai A., Jinnai H. (2021). Cellulose nanofiber-reinforced rubber composites prepared by TEMPO-functionalization and elastic kneading. Compos. Sci. Technol..

[58] Nechyporchuk O., Pignon F., Do Rego A.M., Belgacem M.N. (2016). Influence of ionic interactions between nanofibrillated cellulose and latex on the ensuing composite properties. Compos. Part B Eng..

[59] Kato H., Nakatsubo F., Abe K., Yano H. (2015). Crosslinking via sulfur vulcanization of natural rubber and cellulose nanofibers incorporating unsaturated fatty acids. RSC Adv..

[60] Jiang W., Shen P., Yi J., Li L., Wu C., Gu J. (2020). Surface modification of nanocrystalline cellulose and its application in natural rubber composites. J. Appl. Polym. Sci..

[61] Trovatti E., Carvalho A.J.F., Ribeiro S.J.L., Gandini A. (2013). Simple green approach to reinforce natural rubber with bacterial cellulose nanofibers. Biomacromolecules.

[62] Lee S.H., Md Tahir P., Lum W.C., Tan L.P., Bawon P., Park B.-D., Osman Al Edrus S.S., Abdullah U.H. (2020). A review on citric acid as green modifying agent and binder for wood. Polymers.

[63] Saini S., Belgacem M.N., Bras J. (2017). Effect of variable aminoalkyl chains on chemical grafting of cellulose nanofiber and their antimicrobial activity. Mater. Sci. Eng. C.

[64] Khanjanzadeh H., Behrooz R., Bahramifar N., Wolfgang G., Bacher M., Edler M., Griesser T. (2018). Surface chemical functionalization of cellulose nanocrystals by 3-aminopropyltriethoxysilane. Int. J. Biol. Macromol..

[65] Kargarzadeh H., Sheltami R.M., Ahmad I., Abdullah I., Dufresne A. (2015). Cellulose nanocrystal: A promising toughening agent for unsaturated polyester nanocomposite. Polymer.

[66] Pierre G., Punta C., Delattre C., Melone L., Dubessay P., Fiorati A., Pastori N., Galante Y.M., Michaud P. (2017). TEMPO-mediated oxidation of polysaccharides: An ongoing story. Carbohydr. Polym..

[67] Isogai A., Zhou Y. (2019). Diverse nanocelluloses prepared from TEMPO-oxidized wood cellulose fibers: Nanonetworks, nanofibers, and nanocrystals. Curr. Opin. Solid State Mater. Sci..

[68] Markets and Markets Industrial Rubber Market by Application (Automotive, Building & Construction, Industrial Manufacturing, Polymer Modification, Wire & Cable, Electrical & Electronics, Bitumen Modification), Type, Product, and Region—Global Forecast to 2022. https://www.marketsandmarkets.com/Market-Reports/industrial-rubber-market-42187401.html.

[69] Oksman K. (2012). Nanocelluloses and their use in composite materials. Express Polym. Lett..

[70] Siro I., Plackett D. (2010). Microfibrillated cellulose and new nanocomposite materials: A review. Cellulose.

[71] Roy K., Pongwisuthiruchte A., Debnath S.C., Potiyaraj P. (2021). Application of cellulose as green filler for the development of sustainable rubber technology. Curr. Res. Green Sustain. Chem..

[72] Dominic M., Joseph R., Begum P.S., Kanoth B.P., Chandra J., Thomas S. (2020). Green tire technology: Effect of rice husk derived nanocellulose (RHNC) in replacing carbon black (CB) in natural rubber (NR) compounding. Carbohydr. Polym..

